# Establishment and characterization of a cell line HCS1220 from human liver metastasis of colon cancer

**DOI:** 10.1186/s12935-018-0630-z

**Published:** 2018-09-10

**Authors:** Yi Chai, Huan Wang, Fang Zhou

**Affiliations:** 1 The First Affiliated Hospital of Nanjing Medical University, Nanjing, 210029 Jiangsu China; 2 Key Lab of Drug Metabolism and Pharmacokinetics, China Pharmaceutical University, 24 Tong Jia Xiang, Nanjing, Jiangsu China

**Keywords:** Colon cancer liver metastasis, Cell Line HCS1220, STR analysis, Karyotype analysis, Biomarkers

## Abstract

**Background:**

To establish one primary cell line of human liver metastasis of colon cancer.

**Methods:**

HCS1220 cell line was derived from one liver metastasis of colon cancer patient’s resected tumor sample. The characterization of the cell line was defined by karyotype analysis, short tandem repeat (STR) analysis and mycoplasma contamination. Subcutaneous injection 1 × 10^6^ cells to four BALB/c nude mice, the viable tumors were developed and diagnosed (H&E staining). The expression of biomarkers CK20 and CDX2 for colon cancer were determined by immunocytochemistry assay.

**Results:**

HCS1220 cell line can grow stably and continuously passage. During the grow process, the contact loss in the growth process and superimposed growth, which could be defined as proliferation of malignant tumor. Chromosome analysis revealed the cells derived from human female. The cells were not contaminated by mycoplasma. By immunohistochemistry, the cell line was proven to express the biomarkers of colon cancer CK20 and CDX2, while a-fetoprotein, hep-1 and glypican-3 were stained negative, which demonstrated that the HCS1220 cell line originating from the intestinal tissue.

**Conclusions:**

HCS1220 cell line has the characteristics of primary human liver metastasis of colon cancer. The results of STR have genetically showed that cell line is original, which can provided cell materials for research in vitro and can also help for establishing the mechanism model of liver metastasis of colon cancer and preparing, screening and evaluating anti-tumor drugs.

## Background

Colon cancer is a common clinical malignant digestive system cancer and it is the third malignant in the world [1]. Like other solid tumors, the high mortality of colon cancer is also associated with high metastasis risk and the liver is the major organ [2]. Survey data show that about 15% to 25% of colon cancer patients may occur liver metastases, which is an essential cause of the poor prognosis of colon cancer [3]. Therefore, the control and treatment of liver metastasis is the important method to improve the survival time with colon cancer.

Human primary tumor cell lines are extremely important for studying the pathogenesis of tumors and developing individual therapy. Currently, the tumor cell lines derived from the tumor tissue of patients have been widely used in drug screening and drug resistance research, tumor microenvironment, colon cancer pathogenesis and metastasis mechanism [4]. However, there is few cell line origined from human liver metastasis of colon cancer, just only about 10 human liver metastatic cell lines existing in ATCC, DSMZ, JCRB and RIKEN databases. Therefore, establishing more human liver metastasis of colon cancer cell line is important to tumor research.

In this study, we described the characteristics of human liver metastasis of colon cancer cell line which named HCS1220, including the cell morphology in vitro and short tandem repeat genotyping (STR) [5, 6]. The cell line was not contaminated by mycoplasma. By immunohistochemistry, HCS1220 was proven to express the biomarkers of colon cancer CK20 and CDX2. Subcutaneous injection 1 × 10^6^ cells to four BALB/c nude mice, from the 3rd week, all tumors were formed and poorly differentiated adenocarcinoma in morphology confirmed by histopathological examination. Cytogenetic analysis showed multiple chromosomal aberrations, with a consistent deletion in the long arm and deletions or rearrangements in the short arm of chromosome.

## Methods

### Patient

The cell line was derived from a liver metastasis of colon cancer patient who was a 69-year-old woman in Jiangsu province hospital. 2014 the patient was carried out the surgery of colon cancer. In 2016 Dec, laboratory examination results showed CEA 64.4 ng/ml, CA199 24.4 U/ml, CA724 29.9 U/ml, NSE 32.4 ng/ml; Colonoscopy biopsy showed Sigmoid adenocarcinoma. MRI examination demonstrated a space-occupying lesion in the right lobe. The resected liver tumor was approximately 5 × 5 × 2.5 cm, pathological results showed hepatic adenocarcinoma, II–III stage.

### Establishment of the HCS1220 cell line

A portion of tumor tissue (3 gm) resected from liver during the operation and immediately washed in DMEM medium. The tissue was cut 1 mm^3^ into tissue-culture flasks (Corning Glass Works, Corning, NY), washed twice with l × PBS (Ca^2+^, Mg^2+^ free) and 1 × Wash Medium (Invitrogen), After digestion using 0.1% Collagenase Type IV (Gibco) in DMEM for 30 min at 37 °C and shaked occasionally in a 15 ml centrifuge tube (Corning), the suspension was filtered by 45 μm cell strainer to remove large fragments and the collected liquid was centrifuged consecutively at 1500 rpm, 1000 rpm, 800 rpm and 600 rpm for 5 min, respectively. Cancer cells were resuspended using primary culture medium (DMEM/F12 + 10% FBS + 1% penicillin–streptomycin + 0.2 U/ml insulin) and transferred to a tissue-culture flask overnight in a humidified incubator at 37 °C with 5% CO_2_. Fresh medium can be changed every day.

In the first 3 months, fibroblast cells grew fast. During this time, some tumor cell “island” emerged. We removed the fibroblast scratching by pipetting tips and the tumor cell “island” became bigger. Tumor cell clones were picked out from the primary culture and purified, at passage 40 (13 months after we picked out the tumor cell clone), a stable cell line (HCS1220) was considered to have been established.

### Morphological of HCS1220

HCS1220 were seeded in 25-mm^3^ tissue-culture flasks and incubated at 37 °C in a humidified atmosphere containing 5% CO_2_ for 2 weeks. Everyday the cells were placed under the inverted microscope to observe the general morphology.

### DNA isolation and STR (short tandem repeat) analysis

Genomic DNA from HCS1220 was isolated using genomic extraction kit (Axygen, USA). The cell DNA was amplified by a 20-STR amplification method. STR loci and the sex gene Amelogenin were tested by an ABI 3730XL Genetic Analyzer. The data were analyzed by GeneScan and GeneMapperTM ID Software (Invitrogen).

### Karyotype analysis

We performed chromosomal analysis on HCS1220 cells with low and high passages to determine whether chromosomal abnormalities could be considered in vivo or in vitro phenomena. The cells in logarithmic growth phase were harvested by trypsinization and DMEM/F12 medium were added to stop the reaction. After centrifugation at 1000 rpm for 4 min, 0.56% KCl solution (37 °C) was added and the hypotonic treatment was performed for 30 min. Then the fixative (methanol/acetic acid 3:l) was added to fix the cells twice at room temperature for 30 min. Slides were air-dried and stained with Giemsa stain for 20 min.

### Transplantation

HCS1220 cells were digested with 0.25% trypsin and then counted. After centrifugation at 950 rpm for 3 min, the supernatant was discarded. The cells were resuspend in PBS to prepare a cell suspension of 1 × 10^6^ cells/ml.

Four 6-week-old BALB/c nude mice (Slac Laboratory Animal Company. Ltd, Shanghai) were subcutaneous injected 0.1 ml suspension into the groin and the tumor formation was observed from the 7th day. The mice were sacrificed when tumor burden exceed 10% of the normal body weight. The viable tumor tissues were fixed in 4% formaldehyde, paraffin embedded and diagnosed (H&E staining).

### Detection of biomarkers for xenografts

The expression of biomarkers CK20 and CDX2 for colon cancer and biomarkers AFP, hep-1 and glypican-3 for liver cancer were determined by immunocytochemistry assay. After deparaffinization and antigen retrieval procedure, slides were permeabilized with 0.1% Triton X-100, incubated 5–10 min at room temperature with 0.3% H_2_O_2_ solution and washed with distilled water and soak in PBS twice. Then blocked with 5–10% goat serum (Invitrogen, USA) in PBS at room temperature, after 10 min, The slides were incubated with the following primary antibodies at room temperature overnight: anti-CK20, anti-CDX2, anti-AFP, anti-hep-1 and anti-glypican-3. The secondary antibodies were supplied at 37 °C for 10–30 min. Next horseradish enzyme or alkaline phosphatase labeled streptavidin working solution could be added to incubate at 37 °C for 10–30 min. Finally, the hematoxylin was used in counterstaining.

### Detection of mycoplasma contamination

Myco-PCR-Mix mycophenolate mycoplasma detection kit (Qiao Xinzhou Bioengineering Co. Ltd.) was used to detect mycoplasma contamination.

## Results

### HCS1220 cell morphology

**Fig. 1:**
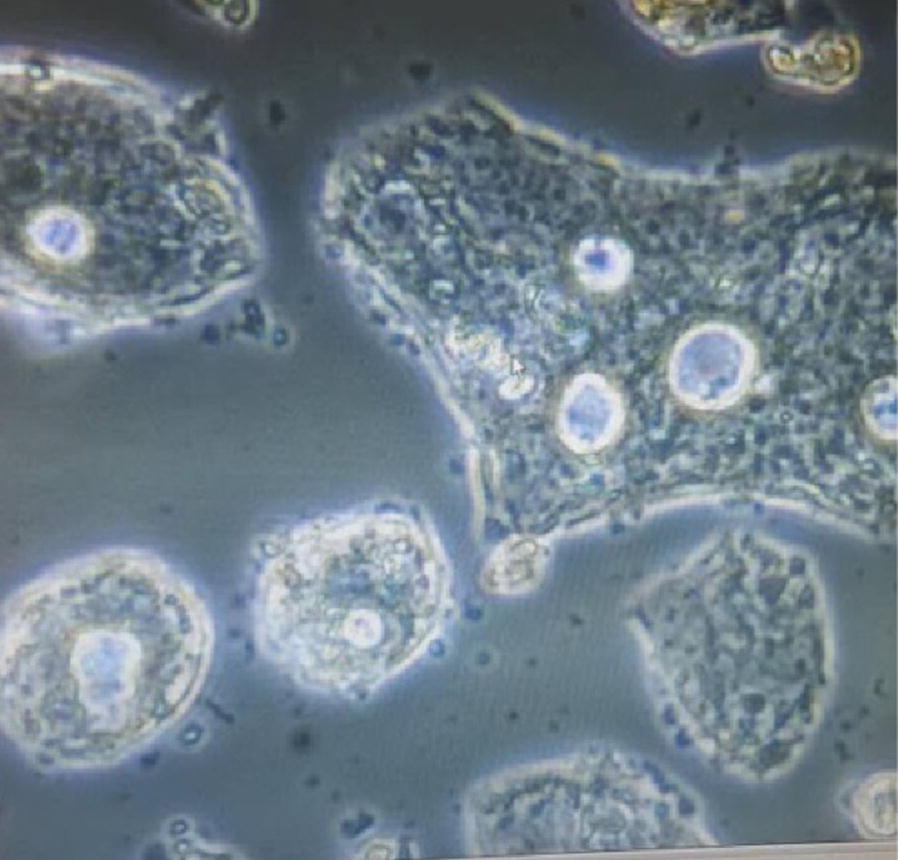
Morphological image of the HCS1220 cells (×400, final magnification)

The HCS1220 cells were obviously apytia with poorly differentiated morphology (Fig. 1). Cells disorderly grew and most of them appeared irregular polygon; Nucleus were larger than normal cells. When these nonadherent cells grew over the whole plate, they would be in overlap and pile up in high density, the results showed the cells had lost touching inhibition and had become malignant cells gradually.

### Analysis of short tandem repeats (STR)

No matched cell lines were found in ATCC, DSMZ, JCRB and RIKEN cell bank and no multiple alleles were detected in the cell line. Test results shown in Table 1.

**Table 1:** DNA fingerprinting analysis using STR loci for newly established HCS1220 cell lines

Loci	Cells to be detected		Cells of the cell bank	
	Cells’ name: 1220-1 A P18		Control cells’ name: 1220-1	
	Allele1	Allele2	Allele1	Allele2
D5S818	10	12	10	12
D13S317	9	9	9	9
D7S820	11	12	11	12
D16S539	9	10	9	10
VWA	17	17	17	17
TH01	9	10	9	10
AMEL	X	X	X	X
TPOX	8	8	8	8
CSF1PO	11	12	11	12
D12S391	19	19	19	19
FGA	25	25	25	25
D2S1338	18	23	18	23
D21S11	29	29	29	29
D18S51	17	17	17	17
D8S1179	13	15	13	15
D3S1358	15	15	15	17
D6S1043	12	19	12	19
PENTAE	16	17	16	17
D19S433	15.2	16.2	15.2	16.2
PENTAD	11	11	11	11

### Chromosome analysis

**Fig. 2:**
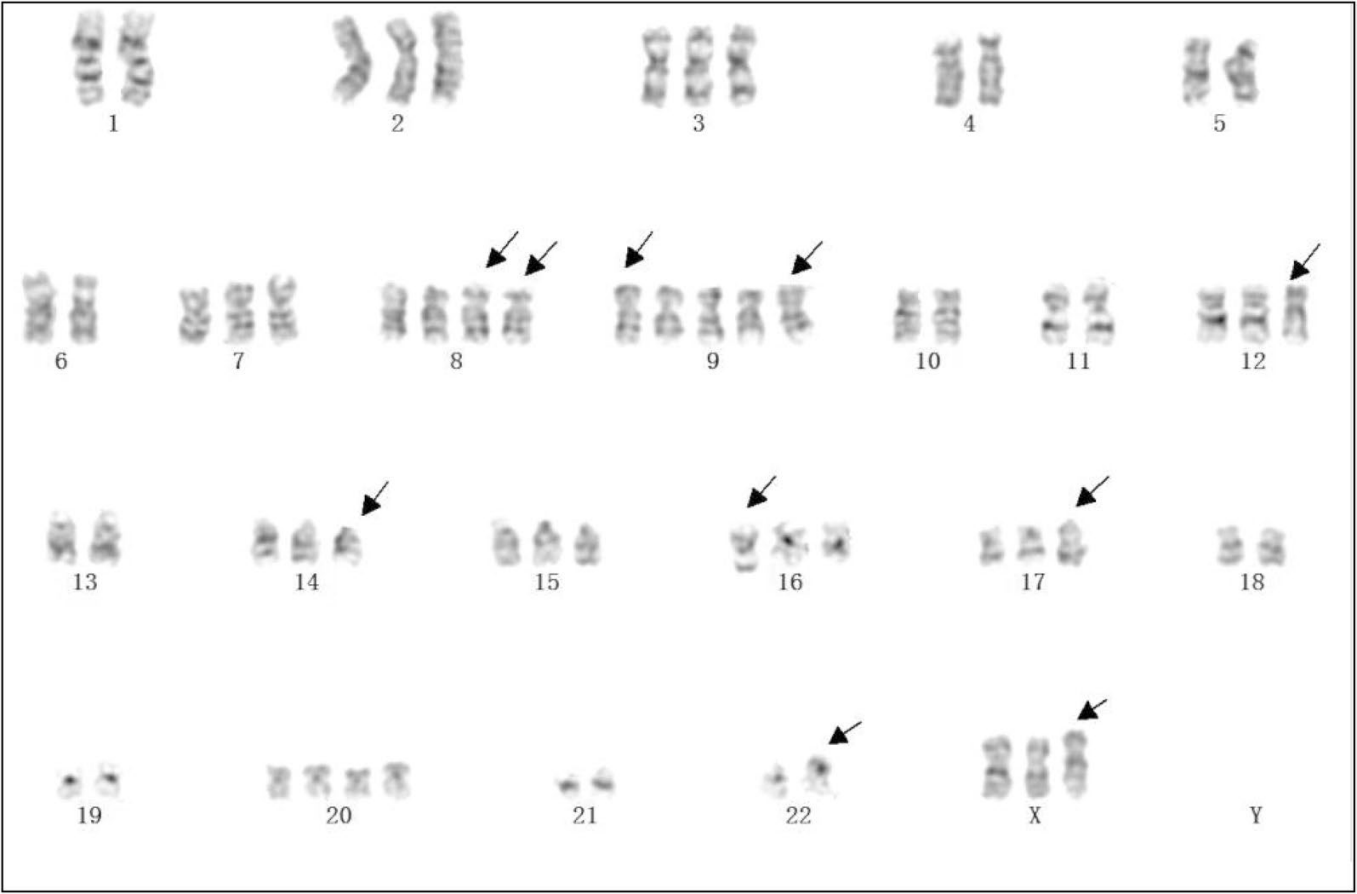
Representative karyotype of HCS1220 cells

The number of chromosomes was distributed in a range of 43–65 with a modal number of 45 (Fig. 2). HCS1220 cells were derived from human female aneuploid cells. The number and structure of chromosomes in the analyzed karyotype cells which abnormal were marked. Malignant proliferation of cells can be characterized as acceleration of nuclear fission, losing of nuclear fragmentation balance, uncontrolling cell proliferation and a substantial increase in gene copy number.

### Mycoplasma contamination

**Fig. 3:**
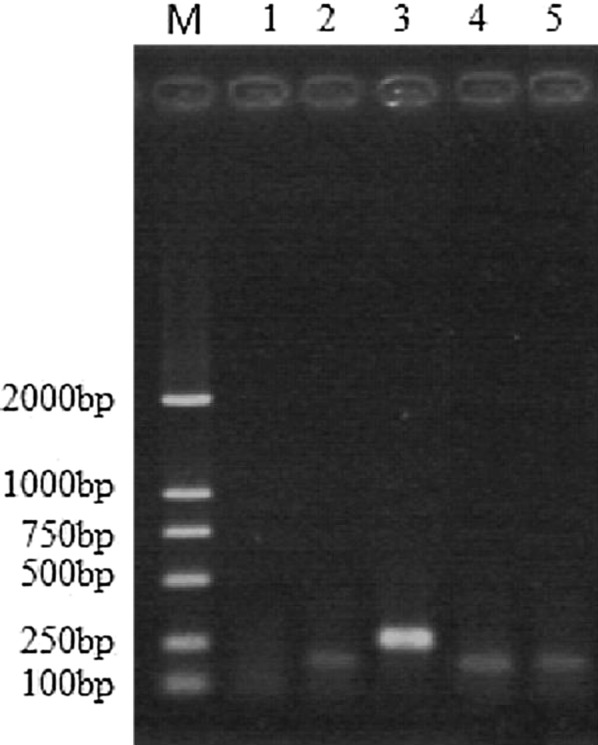
The electrophoretic analysis of mycoplasma contamination M: DNA Marke; Line 1: Negative control; Line 2: Internal Control; Line 3: Positive Control; Line 4 and Line 5: HCS1220

As shown in Fig. 3, the stripe of internal standard control and positive control can be seen at 191 bp, 278 bp, separately. There was only one stripe of the sample at 191 bp, the result showed there was no mycoplasma contamination in HCS1220.

### Xenograft model

**Fig. 4:**
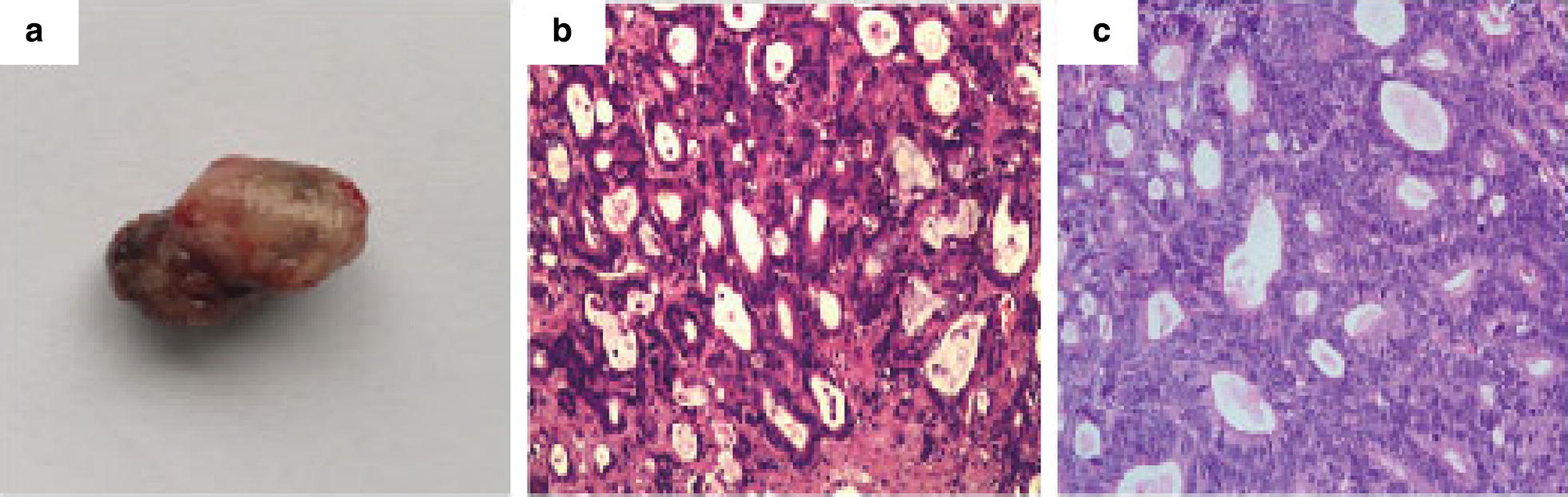
Nude mouse tumorigenicity assay. 1 × 10^6^ HCS1220 cells were subcutaneously injected into the groin of immunodeficient nude mice, after 3 weeks, **a** the solid tumor was obtained. **b** Histological analysis of Clinical tumor specimens and **c** xenografts tumors of nude mice. Sections were stained with haematoxylin and eosin (H&E). Magnification, ×200

Xenograft model was established to confirm the effect of HCS1220 cell on tumor formation in animal. After 3 weeks, all of 4 BALB/c nude mice which subcutaneous injected 1 × 10^6^ HCS1220 cells have formed tumor at the injected sites and some tumors infiltrated the muscle layer, indicating that HCS1220 cells could inform tumor in animal model. Sacrificing the mice and peeling off the tumor, as shown in Fig. 4a, the solid tumor was clearly observed for blood supply and encapsulated. Histopathological results could be seen obviously, such as heterotypic cells type, large and deep staining nuclei and a large proportion of nuclear plasma, what’s more, cells were surrounded by interstitial cells, which formed a large number of duct-like structure (Fig. 4b), the same as the histopathological results of patient’s resected tumor (Fig. 4c). Those were all clinically and pathologically diagnosed as mucinous adenocarcinoma, which was consistent with the pathological features of colon cancer.

### Detection of tumor biomarkers

**Fig. 5:**
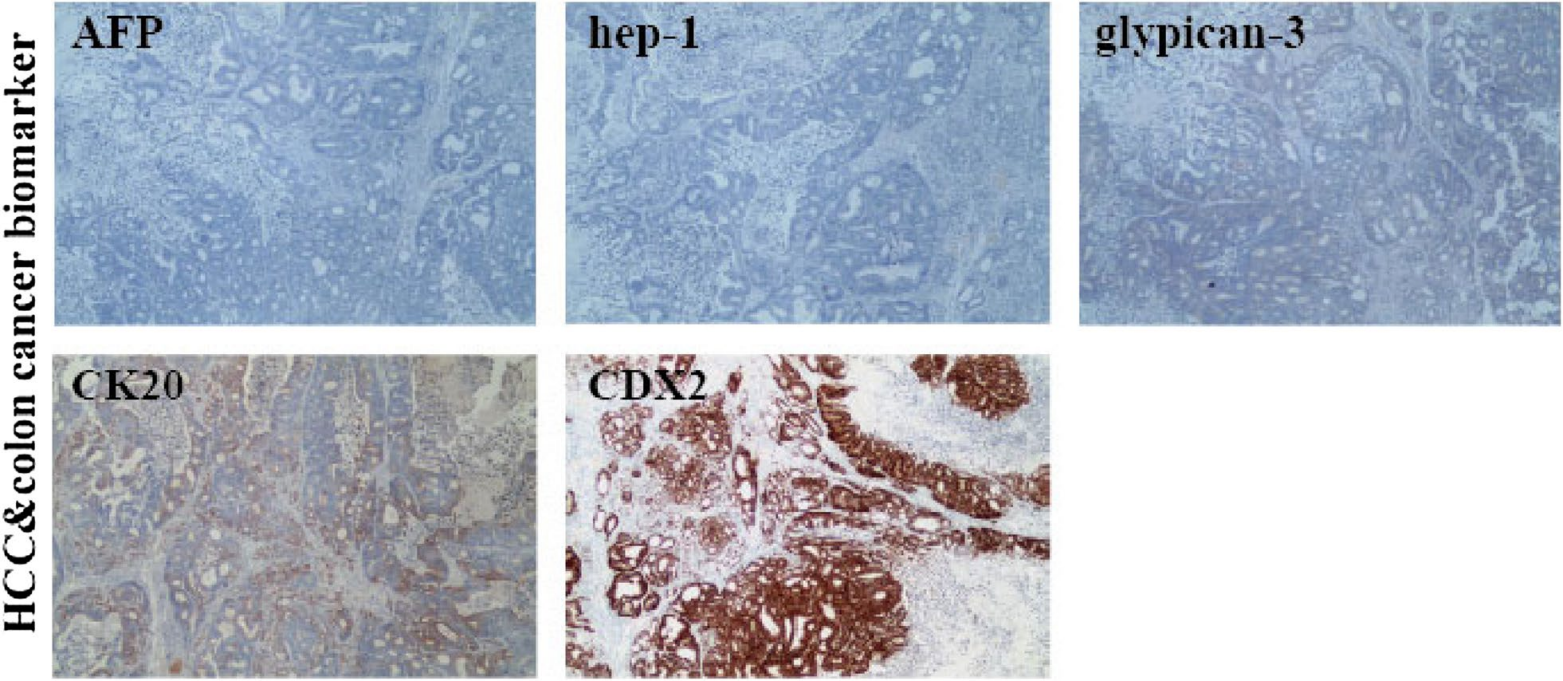
Immunohistochemical stain of the HCS1220 cells. 5 tumor related biomarkers were visualized by special antibodies including colon cancer markers CK20, CDX2 and hepatocellular carcinoma markers AFP, hep-1, glypican-3. Magnification, ×40

The results of immunohistochemical staining for some biomarkers of xenograft tumor were shown in Fig. 5. AFP, hep-1 and glypican-3, the well-used biomarkers for HCC, were negative and two well-known tumor markers for colon cancer, CK20, CDX2 were positive, clearly indicating that xenograft tumor were derived from intestinal tissue.

## Discussion

Most malignant cancers in the liver are not primary but metastasis of cancer from elsewhere in the body, e.g. the colon. However, there are only about 10 human liver metastatic of colon cancer cell lines existing in ATCC, DSMZ, JCRB and RIKEN databases. Establishing sufficient hepatic metastasis of colon cancer cell line is now important to research the mechanism of tumor metastasis, carcinoma growth microenvironment, anti-tumor drugs’ sensitivity influence and the tumor drug resistance.

We have established and characterized a stably growing cell line from a liver metastasis of colon cancer patient which named HCS1220. The cell line cultured in vitro for more than 7 months, at high magnification, HCS1220 has the characteristics of rapid and stable growth, continuous passage, the contact loss during the growth process which could be defined as proliferation of malignant tumor. Chromosome analysis revealed the cells derived from human female. The cells are highly aneuploidy, the number and structure of chromosomes was abnormal. Accelerated nuclear fission, losing the balance of nuclear fragmentation, uncontrolled cell proliferation, a substantial increase in gene copy number, which are signs of polyploid cells, chromosomal abnormalities may be associated with the characteristics of independent of cell growth, the advantages of proliferation and tumorigenicity of the immunocompromised mice.

Cell line identification is strict during development to avoid the risks of using misidentified cells [7]. Recent studies have shown that more than 360 cell lines were cross-contaminated or misidentified without authenticated stock, and the validity of the studies using these cell lines were in doubt [8]. A cell line is considered misidentified when its DNA profile is not consistent with the individual from whom it was derived from. Here, we analyzed that the STR profiles which confirmed that no matched cell lines in 2455 cell line STR data from ATCC, DSMZ, JCRB and RIKEN databases, which means the HCS1220 cell line was first established in the databases. The results also demonstrated the cells were malignant tumor origin [9]. To confirm the origin of the cells, we injecting the cells to establish the implantation model in vitro, the results showed the subcutaneous xenografts of HCS1220 in nude mice were easily informed and presented as expanding outgrowths with strong invasion.

As the tumor resected from the liver, so we explored whether the HCS1220 cell line originated from metastasis of colon cancer or primary liver cancer. From the results of biomarkers assay, we found that no matter the HCS1220 cell or implantation tumor tissue, they all expressed the colon cancer biomarker CK20, CDX2, a-fetoprotein, hep-1, glypican-3 stained negative, the results demonstrated that the HCS1220 cell line originating from the intestinal tissue.

## Conclusion

In summary, HCS1220 cell line has the characteristics of primary human liver metastasis of colon cancer and it is consistent with the established lineage standard. The results of STR has genetically showed that established human tumor cell line from liver metastasis of colon cancer is original, which can provided cell materials for research in vitro in the pathogenesis of liver metastasis and individualized treatment. What’s more, it can also help to provide the foundation for establishing the mechanism model of liver metastasis of colon cancer and preparing, screening and evaluating anti-tumor drugs.

## Abbreviations

STR: short tandem repeat
CK20: cytokeratin
CDX2: caudal type homeobox transcription factor 2
AFP: α-fetoprotein
CEA: carcinoembryonic antigen
CA199: carbohydrate antigen 199
HEP-1: heparinase
CA724: carbohydrate antigen 724
NSE: 2-phospho-d-glycerate hydrolase
MRI: nuclear magnetic resonance imaging
DMEM: Dulbecco’s modified eagle medium
DMEM/F12: Dulbecco’s Modified Eagle Media: Nutrient Mixture F-12
PBS: phosphate buffer saline
FBS: fetal bovine serum
PCR: polymerase chain reaction
H&E: haematoxylin and eosin
DNA: deoxyribonucleic acid
